# Patient Advocacy Group Leaders' Perceptions on Primary Care's Role in Caring for Patients With a History of Breast Cancer

**DOI:** 10.1111/hex.70458

**Published:** 2025-10-07

**Authors:** Kacie Barry, Sarah J. Fadem, Jennifer R. Hemler, Jenna Howard, Lisa Mikesell, Denalee M. O'Malley, Shawna V. Hudson, Benjamin F. Crabtree

**Affiliations:** ^1^ Robert Wood Johnson Medical School Rutgers University Piscataway New Jersey USA; ^2^ Department of Obstetrics, Gynecology and Reproductive Health, Rutgers Health New Jersey Medical School Newark New Jersey USA; ^3^ Department of Family Medicine and Community Health, Robert Wood Johnson Medical School Rutgers University New Brunswick New Jersey USA; ^4^ School of Communication and Information Rutgers University New Brunswick New Jersey USA; ^5^ Institute for Health, Health Care Policy and Aging Research Rutgers University New Brunswick New Jersey USA; ^6^ Rutgers Cancer Institute of New Jersey New Brunswick New Jersey USA

**Keywords:** breast cancer survivor, cancer advocacy groups, cancer survivorship, care transition, patient advocacy, primary care

## Abstract

**Background:**

Cancer advocacy groups engage patients, families and caregivers in navigating the cancer landscape, with a focus on early detection/screening and providing psychosocial and financial support during and after treatment. These groups are influential among their communities, funders and policymakers.

**Objective:**

We wished to understand perceptions of breast cancer advocacy group leaders on primary care's role in breast cancer survivorship care, given limited primary care engagement despite endorsement by the National Cancer Institute (NCI).

**Methods:**

As part of a larger NCI‐funded study, we used purposive sampling to select leaders from a diversity of patient advocacy groups for in‐depth interviews (*n* = 9). After obtaining consent, interviews were conducted and recorded on Zoom, professionally transcribed, and analysed using an established immersion–crystallisation process to identify themes and patterns.

**Results:**

We interviewed leaders (*n* = 9) from two local, three regional and four national advocacy groups, five of whom had personal experiences with breast cancer. These advocates emphasised that transitions away from the safety of oncology to primary care are difficult for patients. They felt patients with a history of breast cancer have unique and complex needs that are different from the standards of care found within primary care settings, and primary care clinicians are not adequately prepared to address these. In reflecting on the ideal role of primary care, they highlighted listening to patients, identifying issues and referring patients to appropriate specialists, but ultimately stressed that patients needed to advocate for themselves in the current healthcare environment.

**Conclusions:**

Advocacy groups typically start as grass root efforts motivated by perceptions of inadequate support and care for cancer patients. As such, there is potential for advocacy groups to shape the conversation to improve collaboration between oncology and primary care by articulating and advocating for better primary care involvement in survivorship care.

**Patient or Public Contribution:**

The project's steering committee included cancer survivors and cancer advocacy group leaders who provided feedback on the project design and made recommendations for people to interview. Steering committee retreats were later held after the completion of interviews to reflect on emerging findings and plan dissemination strategies. The study team included a cancer survivor and several members whose immediate family members had a history of cancer. They were actively engaged in the design, analysis and manuscript writing.

## 1 Introduction

Advocacy organisations promote the well‐being and protect the interests of cancer patients, their families and caregivers. As campaigners for change, they are instrumental in prompting activism for greater cancer research funding and enhancements for cancer screening and treatment [1]. Many cancer advocacy groups specifically focus on cancer research [2], while others aim to impact screening, early diagnosis and treatment [3, 4]. The cancer survivorship movement gained prominence with the establishment of the National Coalition for Cancer Survivorship (NCCS) in 1986 as an organisation known for replacing the words ‘cancer victim’ with ‘cancer survivor’ and advocating for patients to take charge and talk to their doctor [5, 6]. There are now many cancer‐specific advocacy groups [7], with breast cancer advocacy being particularly influential in stimulating research, early screening and detection, and patient awareness.

Despite strong encouragement, the 2006 Institute of Medicine (IOM) report, *From Cancer Patient to Cancer Survivor: Lost in Transition* [8], and efforts of the American Society of Clinical Oncology (ASCO) and the NCCS, gaps persist in cancer patients' long‐term care [9, 10]. A key recommendation of the IOM report was greater engagement of primary care clinicians in this effort; however, patients with a history of cancer remain disengaged from primary care in managing care, except for in some small, specialised practices [11, 12, 13, 14, 15, 16, 17]. Currently, little is known about advocacy groups' perspectives on primary care's role in survivorship care; however, in their role of promoting the well‐being and protection of the interests of cancer patients [1, 3], they could also serve as outside mediators and facilitate the connection between primary care and oncology.

Recent survivorship standards [18] suggest that health system policy should have a framework for the provision of survivorship care that is informed by survivorship stakeholders, as well as relevant guidelines. Perspectives from oncologists [19], primary care clinicians [16, 20] and patients [14, 21] have been explored in an effort to understand why primary care is often disengaged and perceived as lacking the necessary expertise to care for cancer. However, there remains a gap in the literature on the perspectives of advocates, despite their role as a relevant stakeholder in this space and the persistence of suboptimal survivorship care delivery. Given the potential influence of cancer advocacy groups on funding and policy [3, 4], we sought to understand perspectives of leaders from local, regional and national breast cancer advocacy organisations on the role primary care should play in cancer survivorship. Using individual in‐depth interviews, we focus on breast cancer because of the long history of advocacy around breast cancer [3, 4] and the established recommendations for primary care [22, 23].

## 2 Materials and Methods

We conducted virtual individual, semi‐structured interviews with cancer advocacy group leaders between May 2022 and May 2024 as part of an NCI‐funded study focused on primary care's role in caring for people with a history of cancer. Interviews were 30–60 min, with participants being asked open‐ended questions to elicit perspectives on patient breast cancer experiences. Participants received a $30 gift card. The Rutgers University Institutional Review Board approved this study, and participants provided informed consent before being interviewed. We adhered to the Standards for Reporting Qualitative Research guidelines.

### 2.1 Reflexivity

Our team included members with expertise in cancer survivorship research (D.M.O. and S.V.H.), qualitative methods (B.F.C., J.R.H. and J.H.), communication (L.M. and S.F.), and being a medical student (K.B.). One team member had a personal history of cancer, and several had close family members with a history of cancer. None had participated in cancer advocacy groups, so initial understanding about how advocates would respond was limited. Before creating the interview guide, team members met in a safe and collaborative space to share their preconceptions about cancer, cancer survivorship and primary care's role [24]. This group sharing and self‐reflection served as a form of peer‐debriefing and continued throughout data collection and analysis and included many personal experiences with cancer as well as team members' prior work in primary care and on cancer survivorship, so we could understand how it was influencing our interpretation. This process enabled the team to avoid traps of perception and understanding using peer debriefing, particularly to avoid confirmation bias, which is a tendency to interpret new information as confirmation of one's current beliefs [24].

### 2.2 Interview Guide Construction

**Figure 1:**
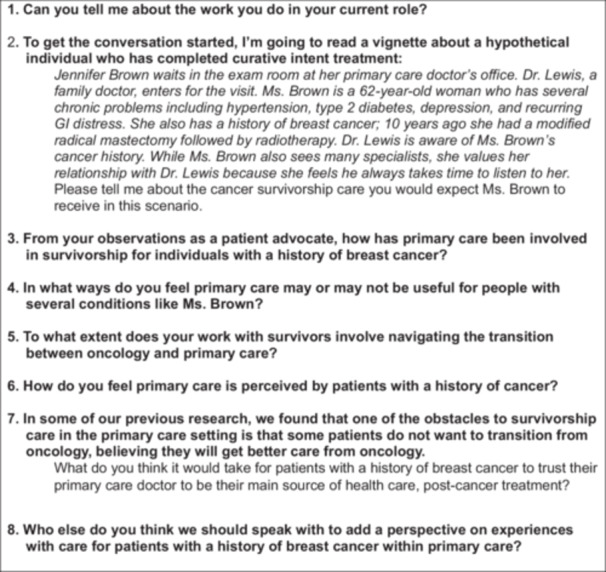
Interview Guide Grand Tour Questions and Vignette.

In creating interview guide questions, team members initially shared and discussed their personal preconceptions of the role of primary care in cancer survivorship to identify possible questions beyond what would be found in the literature [25]. These personal preconceptions were then integrated with the available literature with guidance from our steering committee [25]. To create comparable initial impressions, after an initial ‘ice breaker’ asking about current roles, participants were presented with a vignette of a woman being seen by her family physician (see Figure 1). The vignette was adapted from a clinical story of a typical patient being seen in primary care found in *Doing Qualitative Research*, Chapter 4 [26]. This was followed by open‐ended questions about the roles of primary care and transitions from oncology after completion of treatment. The vignette included common comorbidities (hypertension and diabetes) to mirror what primary care clinicians would typically experience in a visit.

### 2.3 Sampling and Data Collection

We initially conducted four interviews in May and June 2022 based on recommendations of an advisory committee of cancer survivorship experts. After preliminary analysis of early interviews, we conducted online searches and successfully recruited and interviewed five additional participants from regional, statewide‐based advocacy groups and prominent, well‐established national advocacy organisations. This purposeful sampling strategy aimed to attain diversity in organisation type and level of reach, to include local, regional and national organisations. A preliminary analysis of each interview was conducted immediately after completion, after which professional transcriptions were reviewed. Following the analysis meeting for our ninth interview, we determined that the emergent themes were sufficiently robust, and no additional major themes were likely to be identified and thus reached saturation [27].

### 2.4 Data Analysis and Interpretation

After data collection was completed, insights from preliminary analyses were augmented with a thorough analysis of all nine interviews using an immersion–crystallisation organising style [28, 29]. The immersion–crystallisation style is an iterative process that involves alternating periods of deep engagement with the data and reflective analysis, leading to the emergence of themes and insights [28]. Team members met for 1–2‐h meetings dedicated to a single transcript, reading transcripts out loud and sharing insights. Patterns and themes for each interview were crystallised into a table. Once all interviews had been analysed, a comparison of patterns and themes across interviews was conducted to identify overall patterns and themes.

## 3 Results

The nine advocates were women and included leaders from two local, three regional and four national advocacy organisations (Table 1). Interviews averaged 43 min in length. Five advocates had a personal history of breast cancer, which they noted as the impetus for their advocacy work and influenced their professional perspectives. These advocacy leaders described how their organisations were responsive to patients' experiences and needs before diagnosis and during treatment; however, there was little discussion of how their organisations were engaged in long‐term survivorship.

**Table 1:** Description of advocate leaders interviewed.

ID	Cancer survivor?	Type of organisation and description	Years with the organisation	Age range	Self‐described race/ethnicity
1	No	Local non‐profit conducting community breast cancer awareness events	5	20–29	African American
2	No	National non‐profit developing guidelines for follow‐up care of survivors, patient support tools, and continuing education for providers and patients	11	40–49	Non‐Hispanic White
3	Yes	National academic with an interest in research relating to outcomes of cancer survivors and influencing clinical practice guidelines	11	60–69	Non‐Hispanic White
4	Yes	Local non‐profit conducting educational programs and webinars; provides financial assistance and counselling	22	60–69	African American
5	Yes	Regional non‐profit hosting education events with a focus on prevention and diagnosis, providing financial assistance to patient families, and creating a supportive community through an online network	27	70+	Non‐Hispanic White
6	No	Regional non‐profit with a focus on ensuring all women have access to breast cancer screenings, appropriate treatments and follow‐up care	5	40–49	Non‐Hispanic White
7	Yes	Regional non‐profit creating a support network for patients and caregivers in their region through financial assistance and wellness and education resources	7	40–49	Non‐Hispanic White
8	No	National aimed at ensuring organisations meet appropriate standards, obtaining grants related to cancer research and raising awareness of resources available to cancer patients	11	60–69	Non‐Hispanic White
9	Yes	National organisation focusing on overseeing patient navigators who provide support services to patients	5	50–59	African American and White

We identified four themes from depictions of care gaps that these advocacy leaders commonly found among those they serve. For some, these gaps emerged when they put on their patient or caregiver hat and spoke from that experience rather than their advocate role. A particularly compelling gap was about difficulties patients face as they transition from oncology and feel lost or abandoned. All nine tended to be quite positive about primary care overall; however, they perceived patients with a history of breast cancer as having unique and complex needs beyond the standards of care typically provided within primary care. Participants described the ideal activities of primary care as listening to patients, identifying issues and referring patients to appropriate specialists; yet they acknowledged that this ideal role is not yet fully realised. Advocacy leaders placed a strong emphasis on patients with a history of breast cancer needing to advocate for themselves, with advocacy organisations providing resources to do so. We describe these four themes and then discuss potential implications for advocacy organisations in the cancer survivorship landscape and primary care's position within it. Additional supporting quotes for each theme can be found in the supplemental table.

### 3.1 Transitions Away From Oncology to Primary Care Are Difficult for Patients

A recurring sentiment expressed by advocates was that once diagnosed and undergoing treatment, patients find it difficult to let go of their relationship with oncology. They relayed how patients often develop attachments to their oncology team due to the intensity of the cancer experience during active treatment. They noted that patients become disconnected from primary care during active treatment, often describing how consistent interaction with oncology makes patients comfortable with oncology care teams, while primary care takes a back seat. Many advocates did not articulate a clear or consistent role for primary care during active cancer treatment. They described that when patients reached a point of transition out of oncology care, it felt like a blunt transition. At this transition point, patients must either reconnect with their previous primary care provider, who, in many cases, may have been disconnected from their care for several years during their active treatment phase, or try to find a new provider, searching for someone who understands them and has knowledge about a condition that they consider central to their identity. Reflecting on her personal experience with breast cancer, one local advocacy group leader related,

You don't wanna leave the oncologist, but you have to. You feel scared. You feel like you're just dropped because the primary haven't played a big role.

Advocate 4

Another advocate from a major national advocacy organisation explained how the intensity of oncologic care contributes to a patient's strong attachment to oncology providers. In further describing the trust developed with the oncology care team, the founder of a regional advocacy organisation emotionally articulated the power of such a lasting bond,

And I think a lot of that comes out of that trust relationship that's built. This is literally a person who has helped save their life in their mind, right? And so, that does a lot for building a relationship and not wanting to see that severed

Advocate 7

These advocacy leaders described a transition gap, while recognising the need for patients to transition away from oncology at some point post‐cancer treatment.

### 3.2 Advocates Perceive Primary Care as Not Adequately Prepared to Care for the Unique and Complex Needs of Patients With a History of Breast Cancer

These advocates believed that primary care was not adequately prepared to deal with all the details of cancer treatment and survivorship. That is, while primary care clinicians were perceived as having the skills for managing a wide range of acute and chronic issues brought by patients, advocates felt primary care lacked information, education and experience for managing cancer and survivorship. There was a general sense that primary care physicians were reluctant to take on survivorship care because cancer was not their field. As one advocate from a national‐level organisation explained,

I think that primary care is very—primary care physicians are very much focused on sort of the here and the now…. But as far as that extra layer of screening that cancer patients … need and the additional screenings and the additional mindfulness and when red flags go off, in talking to people, I get that sense that that's really not, that [cancer survivors] feel very attached to their oncologist because they know cancer.

Advocate 8

There was a recognition that this was not just a primary care issue, but that this gap was perpetuated by primary care lacking sufficient input from oncology. One advocate and researcher from a national organisation reflected on this point,

Somebody's gotta tell the primary care look, [a survivor] needs an echo every 2 years and she needs an MRI. So those instructions have to be delivered by the oncologist.

Advocate 3

Advocates perceive this gap in preparedness on the part of primary care as stemming from both inadequate training and education regarding cancer and survivorship, as well as a disconnect between the fields of primary care and oncology.

### 3.3 Primary Care Has the Potential to Act as the Central Point for Care for Patients With a History of Breast Cancer

These advocacy leaders recognised that primary care physicians were connectors and coordinators, acknowledging that primary care could play an active role in the care of patients with a history of cancer. One advocacy leader from a local non‐profit organisation noted,

So, first thing that came to mind is like he [primary care physician] can serve in a role as like a connector. So being aware of the different specialists that [the survivor] has to see, those appointments that's needed and serving as like that reminder. Like the one kind of overseeing her overall health as a primary care physician

Advocate 1

This was particularly true about coordinating care with specialists and serving as a ‘captain of your ship’ (Advocate 5). However, there was also a sense that for a patient's breast cancer history, this care coordination role was only aspirational, with advocates stating that they are still seeking primary care doctors who will truly listen to patient concerns within the context of a history of breast cancer. It was noted that this ideal primary care role may be difficult to obtain because some necessary referrals and aspects of cancer go beyond what primary care physicians should be expected to know. However, as a starting point for taking on a care coordination role for these patients, advocates suggested that primary care needs to create the space for patients with a history of cancer to reveal potential issues by listening to patient stories.

So just ask a cancer patient, one, first and foremost, how are you feeling physically and emotionally? Take a few minutes and let them tell their story. Instead of just saying, oh, your blood pressure's good, A1Cs seems good, anything else, let them tell their story first and then go into your exam and what you need to do

Advocate 9

This connector role was seen as being unique to primary care and could be used to the advantage of patients with a history of cancer, by serving as a coordinator of care amidst many involved specialists. Still, there was the recognition that this potential role in the survivorship space was not yet fully realised.

### 3.4 Advocates Perceive Patients With a History of Breast Cancer Need to Advocate for Themselves Due to Existing Gaps in Care

All nine advocates recognised a gap in patients' ability to receive the care they need and to stay informed about their health. Advocates saw part of their organisation's role in supporting patients and learning to proactively advocate for themselves, both during treatment and as cancer survivors. The importance of urgently alerting the care team of any potential issue or concern, whether oncology or primary care, was seen as critical for patients with a history of cancer. As the leader of a regional non‐profit explained,

I always tell everybody you have to be your own best advocate. You only, you know, what your body feels like. If you have a feeling about it, even if the doctor downplays what you're—you know, if you still feel in your gut that something's wrong, that you need to speak up for yourself and say, look, no, I need something more than this

Advocate 5

This need for self‐support was reinforced by the regional office leader of a national advocacy organisation, who noted patients with a history of cancer need to proactively report changes in symptoms and any cancer screening needs,

Some folks never want to talk about cancer again, and understandably, but just really kind of encouraging them to advocate for themselves and make sure, again, that they're notifying their doctors of any new symptoms and staying on top of their kind of screenings and maintenance therapies

Advocate 9

Nevertheless, there was a recognition that self‐advocacy was not realistic for many patients, so these advocacy organisations actively fill gaps in the cancer survivorship care system by providing education and resources while encouraging patients to advocate for themselves.

## 4 Discussion

Cancer advocacy organisations have been instrumental in generating support and funding for cancer research, highlighting screening and early detection, and providing support for cancer patients, families and caregivers [1]. These organisations recognised the unmet needs [9, 10] and issues that patients with cancer faced more than 30 years ago, but new gaps have been exposed related to transitions and survivorship. Our interviews revealed an implicit acknowledgement among advocates that something is wrong in the interface between oncology and primary care. These advocacy leaders recognised a need for eventual transition out of oncology, while acknowledging a failure to successfully transition to primary care clinicians who understand and prioritise a patient's cancer history. This gap raises the question of when and how best to transition patients from oncology.

Because of their unique vantage point, cancer advocacy organisations are in the position to help fix articulated survivorship care gaps on multiple levels: with patients, providers, health systems, and federal and state organisations. Understandably, these advocacy organisations work most closely with oncology, cancer centres, and cancer‐focused public and private organisations like the National Cancer Institute (NCI) and the American Cancer Society. They do not customarily work with primary care organisations. Advocates have an opportunity to educate themselves on primary care abilities and activate patient demand for better primary care supports for survivors that can be used politically to inform healthcare policy decisions. In line with new national standards [18], advocates can help inform the policies of local, state and federal survivorship organisations, to ultimately put pressure on the healthcare system and insurance companies to create and implement transition mechanisms and care coordination between oncology and primary care. Although seemingly a contradiction, patient advocates on one hand believe that primary care lacks all the expertise needed to care for survivors, but on the other hand, they also recognise that primary care has a unique role in serving as a connector. While primary care may lack all the expertise needed, advocacy groups can petition accrediting organisations to provide survivorship care training in medical schools and for primary care residency programs. At the same time, advocacy organisations should reach out to professional organisations such as the Accreditation Council for Graduate Medical Education (ACGME), the National Board of Medical Examiners (NBME) and the Society of Teachers of Family Medicine (STFM) to energise primary care to become more informed and take on more care for patients with a history of cancer.

The small sample of advocates we interviewed is a limitation, and we may have missed some key perspectives. Our sample only included two local and three regional advocacy groups, so it is possible that we may be missing important local and regional differences in healthcare availability and delivery of services. All our respondents were women, some with a personal history of breast cancer and others without this history; however, we did not specifically explore the impact of that history on their perspectives. There was limited ethnic and racial diversity in our sample of advocacy group leaders, with seven of the nine identifying as non‐Hispanic white, which may reflect the population that obtains these leadership positions. Nevertheless, our sample was diverse and purposefully selected [30], and we reached saturation [27] with these nine advocacy group leaders.

Upon reflection, improvements in the gaps outlined above regarding the transition period, preparedness of primary care to manage these patients, and communication between providers and their patients may place the onus back in the hands of the providers, placing less emphasis on patients needing to advocate for themselves due to a more reliably functioning health system. However, until those larger system‐level changes are realised, advocacy groups should continue to serve an important role in empowering and educating patients, while simultaneously pressuring systems to improve.

## 5 Conclusions

Cancer advocacy organisations emerged from a perceived lack of adequate care for cancer patients. While they currently do not identify primary care as a key player in survivorship care, they have the potential to shape the conversation about survivorship in primary care, given the influential role they have with patients, oncology and cancer‐related systems that can be used to define the role of primary care in this space and help this role become realised in current practice.

## Author Contributions

Conceptualisation: Kacie Barry, Sarah J. Fadem, Jennifer R. Hemler, Jenna Howard, Lisa Mikesell, Denalee M. O'Malley, Shawna V. Hudson, Benjamin F. Crabtree. Data curation: Kacie Barry, Sarah J. Fadem, Jennifer R. Hemler, Jenna Howard, Benjamin F. Crabtree. Formal analysis: Kacie Barry, Sarah J. Fadem, Jennifer R. Hemler, Jenna Howard, Benjamin F. Crabtree. Supervision: Benjamin F. Crabtree. Funding acquisition: Shawna V. Hudson, Benjamin F. Crabtree. Writing – original draft: Kacie Barry, Sarah J. Fadem, Benjamin F. Crabtree. Writing –review and editing: Kacie Barry, Sarah J. Fadem, Jennifer R. Hemler, Jenna Howard, Lisa Mikesell, Denalee M. O'Malley, Shawna V. Hudson, Benjamin F. Crabtree.

## Ethics Statement

This study was approved by the institutional review board of Rutgers University (#2021000838). We adhered to the Standards for Reporting Qualitative Research (SRQR) reporting guidelines.

## Consent

All participants provided verbal consent.

## Conflicts of Interest

The authors declare no conflicts of interest.

## Supporting information


**Table 2:** Thematic Summary Table.

## Data Availability

The data that support the findings of this study are available on request from the corresponding author. The data are not publicly available due to privacy or ethical restrictions.
