# Supplementary material for: Patient Advocacy Group Leaders' Perceptions on Primary Care's Role in Caring for Patients With a History of Breast Cancer
Source: Health Expect. 2025 Oct 7;28(5):e70458. doi: 10.1111/hex.70458 (PMC12501495; doi:10.1111/hex.70458)
Supplement: Supplementary file 1 — Table 2: Thematic Summary Table. [file HEX-28-e70458-s001.pdf]

Table 2. Thematic Summary Table

Theme	Representative Quote
Transitions away from oncology to primary care are difficult for patients.	*You don't wanna leave the oncologist, but you have to. You feel scared. You feel like you're just dropped because the primary [primary care provider] haven't played a big role. (Advocate 4)*
	And I think a lot of that comes out of that trust relationship that's built. This is literally a person [oncologist] who has helped save their life in their mind, right? And so, that does a lot for building a relationship and not wanting to see that severed. (Advocate 7)
	Yeah, some people think like, I'm being kicked out the door after all this fabulous care that I've been getting on one-on-one. (Advocate 8)
	Because it was difficult, those first few months [of transition]. You really felt like you were just in a no-man's land because you were so used to doctors seeing you every week and poking and prodding you, and now no one's talking to you. So it could create some of, just, again, that feeling of loneliness, honestly, or it could create some anxiety or depression. Fear of recurrence is big in survivorship. (Advocate 9)
	Even from my personal experience, at least for me, I tend to ask my oncologist now, regular stuff, and she'll be like, you need to talk to your primary care provider because I got really comfortable with my oncologist. (Advocate 9)
Advocates perceive primary care as not adequately prepared to care for the unique and complex needs of patients with a history of breast cancer.	*Somebody's gotta tell the primary care look, [a survivor] needs an echo every two years and she needs an MRI. So those instructions have to be delivered by the oncologist. (Advocate 3)*
	I know that there's been a movement to push it to primary care, and primary care physicians are overworked. They're worried about everything else, diabetes, heart disease. I mean, there's so much that they're – how can we expect a primary care physician to know that a survivor of Hodgkin's that was radiated 20 years ago is at risk for – how can we expect them to know that? (Advocate 3)
	As a cancer survivor, I can't go to a place and find a trusted primary care physician who's going to really have a lot of experience in what I'm dealing with. And isn't that – no matter what your condition is, don't we all want to go to a doctor who has dealt with a lot of that, right? Don't we all want to find the expert, the best expert we can for what we're dealing with? (Advocate 7)

	… people [physicians] have their specialties, and I think that some people just don't have kind of a lot of in-depth information about what needs to be done. (Advocate 8)
	I think that primary care is very – primary care physicians are very much focused on sort of the here and the now… But as far as that extra layer of screening that cancer patients [need]… and the additional mindfulness and when red flags go off, in talking to people, I get that sense that that's really not, that [cancer survivors] feel very attached to their oncologist because they know cancer. (Advocate 8)
Primary care has the potential to act as the central point for care for patients with a history of breast cancer.	*So, first thing that came to mind is like he [primary care physician] can serve in a role as like a connector. So being aware of the different specialists that [the survivor] has to see, those appointments that's needed and serving as like that reminder. Like the one kind of overseeing her overall health as a primary care physician. (Advocate 1)*
	That's all you want as a primary doctor that will listen to you. And maybe he can pick up something that you're saying that he may think is serious and you may not think is serious. Something like…I'm getting this back pain right here in my back. You need a primary doctor that's gonna say, okay, listen, all right, she had breast cancer, let me move on this. It may be something. I'm just trying to look for a primary doctor that will listen. (Advocate 4)
	I know like even in my own family, like my brother has like conditions and he goes to see his, you know, specialist, but at the helm is the primary care physician, you know, because you need a captain of your ship. (Advocate 5)
	I'm not sure what roles, but maybe it [the role of primary care] would be the point person to communicate and to understand and take that more holistic view of everything that's going on…I mean, you would hope that they'd be the comprehensive point person, but it's mostly communication between all of those doctors where things start to fall through. (Advocate 6)
	I would hope that he [primary care provider] would be on top of all her screenings, that he would listen to her [the survivor's] concerns and take them seriously. I'm sure that, like I'm saying, the GI issues, her blood pressure issues, keeping an eye, does she need a cardiologist? She said she had many specialists, but has she been – his role might also be, have

	you seen your cardiologist lately? Have you seen your GI, if you have a GI? If you haven't, when was the last time you had a colorectal cancer screening? And to make sure that she is up to date on everything, her vaccinations and everything else as well. (Advocate 8)
	So just ask a cancer patient, one, first and foremost, how are you feeling physically and emotionally? Take a few minutes and let them tell their story. Instead of just saying, oh, your blood pressure's good, A1Cs seems good, anything else, let them tell their story first and then go into your exam and what you need to do. (Advocate 9)
Advocates perceive patients with a history of breast cancer need to advocate for themselves due to existing gaps in care.	*So right now many survivors receive the same level of care or those who are the loudest, the most aware of their needs, the most empowered to ask for more support receiving the highest level of care even though medically they oftentimes are the ones that don't need that high level of resource intensive care. (Advocate 2)*
	If everybody that got cancer was able to be empowered and be their own advocate, that would be great. But the reality is not all survivors are able to do that. So again, I believe oncology owns this problem. But until they're incentivized to solve it, we're gonna be in this situation, I believe. (Advocate 3)
	It's no longer where you put all the weight on the primary doctor, especially once you've been diagnosed with cancer, you gotta play a part. Your doctor don't have a little Rolodex with your name and all your little problems on it. You gotta bring it to his attention. So that's what we try to tell you. You gotta empower yourself, you know? (Advocate 4)
	I always tell everybody you have to be your own best advocate. You only, you know what your body feels like. If you have a feeling about it, even if the doctor downplays what you're - you know, if you still feel in your gut that something's wrong, that you need to speak up for yourself and say, look, no, I need something more than this. (Advocate 5)
	And with my parents and my in-laws dealing with their healthcare as they age, and all of these little problems crop up, is they don't really have a patient advocate. They only have themselves. (Advocate 6)
	Some folks never want to talk about cancer again, and understandably, but just really kind of encouraging them to advocate for themselves and make sure, again, that they're notifying their doctors of any new symptoms and staying on

	top of their kind of screenings and maintenance therapies. (Advocate 9)
